# Hydatidiform Mole and coexisting fetus following intrauterine insemination: a case report

**DOI:** 10.5935/1518-0557.20250011

**Published:** 2025

**Authors:** Cynthia Lopes Pereira de Borborema, Eduardo Oliveira Pacheco, Aley Talans, Lucas Rios Torres, Angela Hissae Motoyama Caiado, Felipe Lazar Junior, Ulysses dos Santos Torres, Giuseppe D’Ippolito

**Affiliations:** 1 Abdominal Radiology Department, Grupo Fleury, São Paulo, (São Paulo), Brazil; 2 Clínica Lazar, São Paulo, (São Paulo), Brazil

**Keywords:** hydatidiform mole, assisted reproductive techniques, magnetic resonance imaging

## Abstract

Gestational trophoblastic diseases (GTD) comprise a heterogeneous group of disorders arising from genetic anomalies occurring during fertilization in twin pregnancies and often may be associated with assisted reproductive techniques. An exceedingly rare presentation of GTD is a twin pregnancy hydatidiform mole with a co-existing fetus, condition which may be an important cause of complications for the mother and the fetus. A 36-year-old woman (G2, P0, A1) underwent a friendly controlled ovarian stimulation (COS) followed by intrauterine insemination (IUI) for assisted reproductive purposes, resulting in a twin pregnancy initially characterized by two gestational sacs. However, one sac failed to progress and instead degenerated into molar trophoblastic disease, while the other sustained a normal fetus with regular growth. At 33 weeks gestation, the patient developed preeclampsia, necessitating delivery via cesarean section at a tertiary care facility. Reproductive-assisted procedures may be linked to cases of trophoblastic disease. Additionally, the presence of cystic lesions warrants a wide differential diagnosis, with magnetic resonance imaging serving as a valuable tool for accurate assessment and differentiation of structures.

## INTRODUCTION

Gestational trophoblastic diseases are a heterogeneous group of disorders that occur in twin pregnancies and result from a genetic anomaly that arises during fertilization (Sánchez-Ferrer *et al.,* 2013). It’s characterized by abnormal proliferation of the trophoblast and can be either a partial or complete hydatidiform mole (CHM). The complete form can exist alongside a viable fetus and a normal placenta (Godinho *et al.,* 2014; Zilberman Sharon *et al.,* 2019). It’s a rare condition, with an estimated incidence ranging from one per 20,000-100,000 pregnancies (Nobuhara *et al.,* 2018; Zilberman Sharon *et al.,* 2019).

Molar pregnancies may also happen following assisted reproductive techniques such as in vitro fertilization (IVF) and intracytoplasmic sperm injection (ICSI). In such instances, the incidence of molar pregnancy is estimated to be around 20 per 100.000 pregnancies (Alpay *et al.,* 2021).

In addition, it’s a potential cause of complication for the mother and the fetus including vaginal bleeding, hyperthyroidism, preeclampsia, fetal malformation, death or fetal growth restriction, preterm delivery, hyperemesis, and fetal-maternal hemorrhage (Lin *et al.,* 2021). Moreover, there is an elevated risk of gestational trophoblastic neoplasia (GTN) which varies from 16% to 50% (Giorgione *et al.,* 2017).

Over the past two decades, slightly more than two hundred cases of complete hydatidiform moles with a coexisting fetus have been documented in the literature (Mora-Palazuelos *et al.,* 2023) with a limited number of studies that have focused on diagnostic methods and differential diagnosis (Wang *et al.,* 2023).

In this way, magnetic resonance is a useful diagnostic imaging method because it may assess important structures for the differential diagnosis such as myometrial and parametrial involvement and it can visualize the two amniotic sacs and a normal placenta (Gajewska *et al.,* 2020).

## CASE DESCRIPTION

**Figure 1:**
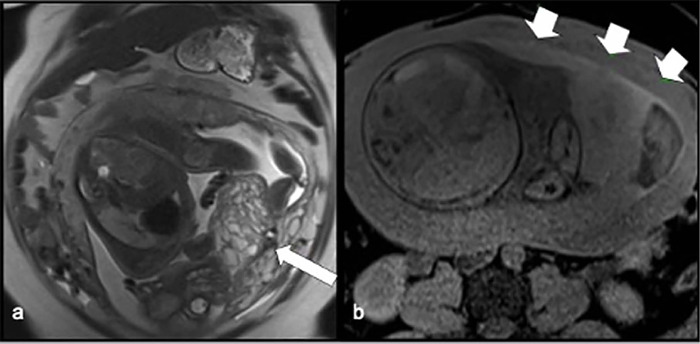
Coronal T2-WI (a) presenting fetus with normal development associated with a multi-cystic mass inferiorly and Axial T1-WI fat-saturated imaging (b) shows a retroplacental hemorrhage.

**Figure 2:**
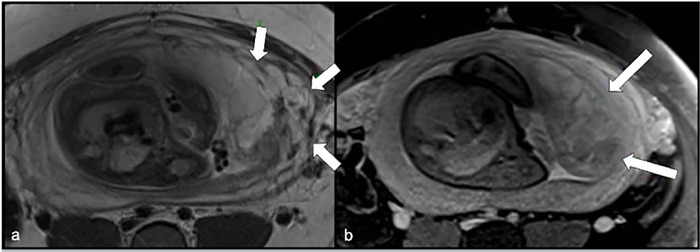
Axial (a) T2-weighted and (b) T1-weighted images. Areas of an undefined plane between the myometrium and the placental bed.

A 36-year-old woman (G2, P0, A1) underwent a friendly controlled ovarian stimulation (COS) followed by intra-uterine insemination (IUI) for assisted reproductive purposes, resulting in a twin pregnancy characterized by two gestational sacs. One of them has not evolved and degenerated into a molar trophoblastic disease. The other was a normal fetus presenting with regular growth. She was referred to our service at 27 + 2 weeks pregnancy to do an MRI evaluation (Fig. 1 and 2) with concerns about complications, such as an invasive mole with a high risk for massive bleeding during labor, and a possibility to evolve into a hysterectomy. Her history included a prior spontaneous abortion due to a genetic disorder (22 Trisomy) with a need for curettage.

The MRI findings indicated the presence of a multi-cystic mass adjacent to the left anterolateral uterine wall, along with a normal placenta positioned cranially, which is indicative of a hydatidiform mole and coexisting fetus (Fig. 1a). Additionally, there was a thin hemorrhagic layer related to retroplacental hemorrhage (Fig. 1b) and regions of indistinct plane between the myometrium and the placental bed, suggesting a possible infiltration plane (Fig. 2).

**Figure 3:**
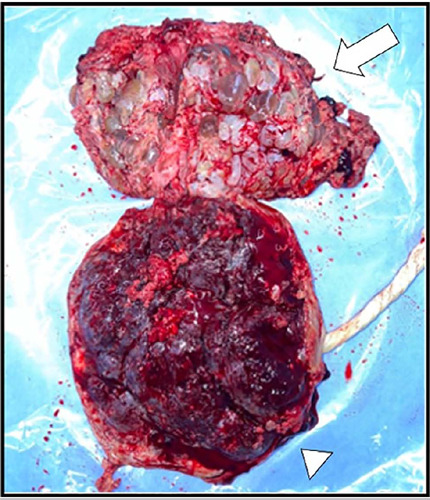
Following the cesarean section, the macroscopic appearance revealed molar tissue (arrow) and a normal placenta (arrowhead), confirming the diagnosis of a hydatidiform mole with a co-existing fetus.

After the diagnosis, the couple decided to continue the pregnancy because of the absence of maternal symptoms. However, she developed preeclampsia at 33 weeks. It was decided to end the pregnancy at 36 weeks as the patient presented with poor blood pressure control and a need for magnesium sulfate. She was submitted to a cesarean section in a tertiary center (Fig. 3). The delivery occurred without complications and the team opted against performing a hysterectomy due to the patient’s desire to preserve fertility. After 5 days, both the mother and the newborn were discharged from the hospital with stable conditions and the patient continued to use anti-hypertensive medications.

## DISCUSSION

Since the advent of assisted reproductive techniques (ART), twin pregnancies have been increasing and can be associated with a higher risk of perinatal morbidity and mortality (Zilberman Sharon *et al.,* 2020). One of these conditions is gestational trophoblastic disease which comprises a wide spectrum of diseases such as the hydatidiform mole (complete or partial), invasive mole, choriocarcinoma, and placental site trophoblastic tumor (PSTT) (Lurain, 2010).

CHM occurs subsequently to the fertilization of an enucleated egg by either two spermatozoa or a haploid spermatozoon, which undergoes duplication, leading to the formation of a diploid conception. A partial hydatidiform mole (PHM) typically has a triploid origin. It emerges when a haploid ovum is fertilized by a single spermatozoon that duplicates, or when two sperm cells fertilize a haploid egg (Alpay *et al.,* 2021).

Hydatidiform pregnancy is linked to various risk factors, with certain studies indicating a potential correlation with fertility treatments. However, the absence of sufficient data prevents making definitive conclusions regarding this relationship. A previous hydatiform mole is considered the primary risk factor and can increase the likelihood of another hydatidiform conceptus tenfold. Extremes of age are also commonly recognized as risk factors, with women aged over 35-40 years facing a 2-to-7.5-fold increased risk for a molar conceptus, respectively (Zilberman Sharon *et al.,* 2019).

Molar pregnancy may occur in ART pregnancies, and the incidence may also be higher in frozen cycles compared to fresh ones (Nickkho-Amiry *et al.,* 2019). One explanation for complete mole formation following ICSI procedures may be superovulation, which can lead to the development of enucleated eggs. Other factors comprise the loss of maternal chromosomal material, disruptions to the meiotic spindle during oocyte manipulation, or because of oocyte fragmentation or degeneration (Alpay *et al.,* 2021).

Prenatal diagnosis of gestational trophoblastic disease (GTD) may include ultrasound (US), magnetic resonance imaging evaluation, serum hCG level, and cytogenetic analysis of the fetoplacental karyotype (Suksai *et al.,* 2017; Wang *et al.,* 2023). US evaluation is considered a reliable tool for the diagnosis at the end of the first trimester (Sánchez-Ferrer *et al.,* 2013; Giorgione *et al.,* 2017; Lin *et al.,* 2019).The characteristic findings include a complex cystic pattern exhibiting a “snowstorm” appearance, separated from a normal placenta (Giorgione *et al.,* 2017).

Although placental or pelvic MRI does not have a routine role in diagnosing GTD, it’s a reasonable option to increase confidence and improve the diagnosis and treatment, especially in cases involving atypical presentations, recurrences, or PSTT/ epithelioid trophoblastic tumor (Lin *et al.,* 2019). MR imaging offers several advantages as it uses nonionizing radiation, provides a large field of view, delivers good tissue contrast resolution, and is operator-independent (Imafuku *et al.,* 2018).

MRI is not associated with an increased risk of harming the fetus. It’s capable of obtaining a whole image of the uterus, especially in cases where the relationship between a mole, and normal placenta is difficult to determine in the US, leading us to a more precise diagnosis (Imafuku *et al.,* 2018). When there is a suspicion of GTN, MRI can play an important role in identifying the anatomic stage of the disease, localizing the tumor, evaluating vascularization, and showing extension through the myometrium, adjacent pelvic organs, and asses pelvic lymph node status (Shanbhogue *et al.,* 2013; Shaaban *et al.,* 2017).

Both CHM and PHM can be normal or show little abnormalities in the first trimester at MRI imaging. The tumor may be visualized as an expansible heterogeneous mass distending the uterine cavity with a “cluster of grapes appearance”. During this period, its high signal intensity on T2-weighted images reflects the vesicular nature of the tumor (Allen *et al.,* 2006).

In the second trimester, it’s possible to find small internal cysts within the mass on T2-weighted images. Myometrium can also be visualized as a hypointense layer surrounding the molar tissue. There is a sharp and smooth distinction in the interface between the mass and myometrium. Additionally, there are dilated vessels represented as signal voids demarcating tumor neovascularity and arteriovenous shunt. On T1-weighted imaging, focal hyperintensity can be interpreted as areas of hemorrhage. After contrast media administration the mass has a heterogeneous enhancement (Herek & Karabulut, 2013; Shaaban *et al.,* 2017).

An unusual presentation of GTD is a twin pregnancy hydatidiform mole with a co-existing fetus (HMCF). One of the main differential diagnoses relies on placental mesenchymal dysplasia (PMD) because both entities can present with enlarged cystic-appearing placenta in ultrasound (Himoto *et al.,* 2014; Marusik *et al.,* 2017). Other differential diagnoses may include PHM with a fetus, placental chorioangioma, intraplacental hemorrhage, and confined placental mosaicism (Giorgione *et al.,* 2017; Suksai *et al.,* 2017).

PMD is a rare vascular anomaly, occurring in 0.02% of all pregnancies. However, it often happens in normal karyotype fetuses, it can also be associated with Beck-with-Wiedemann syndrome. There is a higher prevalence among females, with a 1:3.6-8 male-to-female ratio. The condition is marked by enlargement of the placenta, as well as dilation and congestion of the vessels within the chorionic plate, accompanied by edema in the stem villi and no signs of trophoblastic proliferation (Himoto *et al.,* 2014; Marusik *et al.,* 2017).

Alternatively, to ultrasound evaluations, MRI is especially important in those cases because it can help distinguish the two conditions. Unlike PMD characterized by singleton pregnancy, HMCF consists of two distinct sacs: one contains the fetus and its normal placenta, while the other contains the molar tissue (Shaaban *et al.,* 2017).

## CONCLUSION

This case exemplifies that reproductive-assisted procedures may be linked to cases of trophoblastic disease. Also, there is a wide differential diagnosis of cystic lesions that are not so commonly known by the multidisciplinary team, and it must include placental mesenchymal dysplasia and twin pregnancy hydatidiform mole with a co-existing fetus. Thus, MRI is important in those cases as it can aid in distinguishing the two conditions as it has a large field of view and good tissue contrast without harming the viable fetus.
